# Effect of Sodium-Glucose Cotransporter 2 Inhibitors for Heart Failure With Preserved Ejection Fraction: A Systematic Review and Meta-Analysis of Randomized Clinical Trials

**DOI:** 10.3389/fcvm.2022.875327

**Published:** 2022-05-04

**Authors:** Hufang Zhou, Wenhua Peng, Fuyao Li, Yuelin Wang, Baofu Wang, Yukun Ding, Qian Lin, Ying Zhao, Guozhong Pan, Xian Wang

**Affiliations:** ^1^Institute of Cardiovascular Diseases, Dongzhimen Hospital, Beijing University of Chinese Medicine, Beijing, China; ^2^Jinan Municipal Hospital of Traditional Chinese Medicine, Shandong University of Traditional Chinese Medicine, Jinan, China; ^3^Changping District Hospital of Integrated Traditional Chinese and Western Medicine, Beijing, China; ^4^Dongfang Hospital, Beijing University of Chinese Medicine, Beijing, China

**Keywords:** sodium-glucose cotransporter 2 inhibitors, heart failure with preserved ejection fraction, randomized controlled trials, systematic review, meta-analysis

## Abstract

**Background:**

Heart failure with preserved ejection fraction (HFpEF) is associated with a high risk of mortality and frequent hospitalization. Sodium-glucose cotransporter 2 (SGLT2) inhibitors have favorable cardiovascular protective effect and could decrease the risk of mortality and hospitalization in patients with heart failure with reduced ejection fraction. However, the effect of SGLT2 inhibitors for HFpEF has not been well studied.

**Purpose:**

The aim of this meta-analysis is to systematically assess the effects of SGLT2 inhibitors in patients with HFpEF.

**Methods:**

MEDLINE, EMBASE, Ovid, Cochrane Library, Chinese National Knowledge Infrastructure Database, VIP database, Chinese Biomedical Database, and Wanfang Database were searched from inception to November 2021 for randomized controlled trials (RCTs) of SGLT2 inhibitors for HFpEF. Risk bias was assessed for included studies according to Cochrane handbook. The primary outcome was the composite of first hospitalization for heart failure (HHF) or cardiovascular mortality. First HHF, cardiovascular mortality, total HHF, all-cause mortality, exercise capacity, ventricular diastolic function, and adverse events were considered as secondary endpoints. PROSPERO registration: CRD42021291122.

**Results:**

A total of 12 RCTs including 10,883 patients with HFpEF (SGLT2 inhibitors group: 5,621; control group: 5,262) were included. All included RCTs were at low risk of bias. Meta-analysis showed that SGLT2 inhibitors significantly reduced the composite of first HHF or cardiovascular mortality (HR:0.78, 95% CI: [0.70, 0.87], *P*< 0.00001, *I*^2^ = 0%), first HHF (HR:0.71, 95% CI: [0.62, 0.83], *P* < 0.00001, *I*^2^ = 0%), total HHF (RR:0.75, 95% CI: [0.67, 0.84], *P*<0.00001, *I*^2^ = 0%), E/e’ (MD: –1.22, 95% CI: [–2.29, –0.15], *P* = 0.03, *I*^2^ = 59%) and adverse events (RR:0.92, 95% CI: [0.88, 0.97], *P* = 0.001, *I*^2^ = 0%). No statistical differences were found in terms of cardiovascular mortality, all-cause mortality, NT-proBNP, BNP and 6-min walk test distance.

**Conclusion:**

SGLT2 inhibitors significantly improve cardiovascular outcomes with a lower risk of serious adverse events in patients with HFpEF. However, these findings require careful recommendation due to the small number of RCTs at present. More multi-center, randomized, double-blind, placebo-controlled trials are needed.

**Systematic Review Registration:**

[https://www.crd.york.ac.Uk/prospero/], identifier [CRD42021291122].

## Graphical Abstract

A systematic review and meta-analysis was performed to investigate the effect of sodium-glucose cotransporter 2 inhibitors for heart failure with preserved ejection fraction.

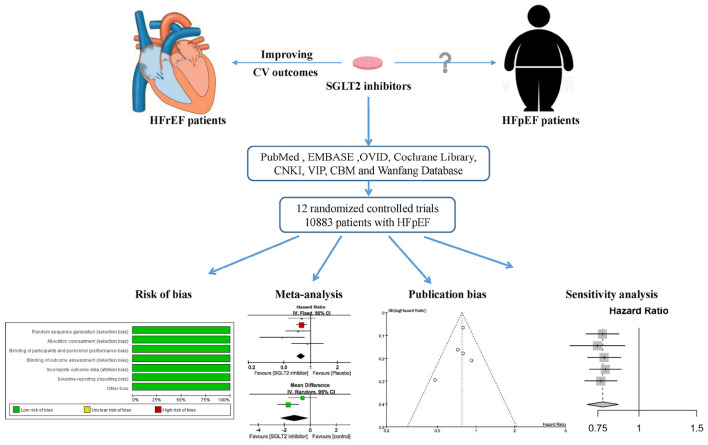

## Introduction

Heart failure with preserved ejection fraction (HFpEF) has been recognized as an important phenotype of heart failure based on the measurement of left ventricular ejection fraction (1). Currently, the prevalence of HFpEF exceeds 8% in people over 65 years of age, accounting for more than 50% of all patients with heart failure (2–4), and its prevalence increases as the population ages (5). Studies have shown that HFpEF is associated with a higher risk of mortality and frequent hospitalization relative to heart failure with reduced ejection fraction (HFrEF) (6–8). HFpEF has become a serious public health problem and has brought a huge economic burden to society (9, 10). However, in sharp contrast, there is a lack of effective drugs for the treatment of HFpEF. According to the 2021 ESC guidelines for the diagnosis and treatment of acute and chronic heart failure (1), no treatment has been shown to convincingly reduce mortality and morbidity in patients with HFpEF, though some specific phenotypes of patients within the overall HFpEF umbrella have shown improvements. These include angiotensin converting enzyme inhibitors (11), angiotensin receptor blocker (12, 13), spironolactone (14), digoxin (15), and sacubitril/valsartan (16).

Studies have shown that patients with type 2 diabetes are at increased risk of developing HFpEF, and there is a higher risk of mortality in patients who have both type 2 diabetes and HFpEF (17–20). Sodium-glucose cotransporter 2 (SGLT2) is the main transport protein responsible for the reabsorption of glucose in the kidneys (21). SGLT2 inhibitors increase urinary glucose excretion to reduce serum glucose by blocking glucose reabsorption in the proximal tubules of the kidney (22, 23). In particular, several large, placebo-controlled clinical studies have shown that SGLT2 inhibitors have beneficial cardiovascular and renal protective effects independent of blood glucose reduction, and could reduce the risk of death and hospitalization in patients with HFrEF (24–26). The cardio-renal protective mechanisms of SGLT2 inhibitors remain incompletely understood, but they are thought to be related to diuretic and natriuretic effects, attenuation of cardiac inflammation and fibrosis, reduction of oxidative stress, reduction of arterial stiffness, improved endothelial function, blood pressure reductions, and reduction in left ventricular (LV) preload and afterload (27, 28). Given its cardiovascular protective effects, SGLT2 inhibitors are recommended in the latest clinical practice guidelines as a cornerstone drug for the treatment of HFrEF (1). However, the effect of SGLT2 inhibitors in patients with HFpEF has not been well studied. Although several recent large-scale trials have explored the effect of SGLT2 in the treatment of HFpEF, unfortunately these results are inconsistent (29, 30). Until now, there has been no systematic review on the efficacy and safety of SGLT2 inhibitors for HFpEF. Therefore, the purpose of this systematic review and meta-analysis is to systematically assess the efficacy and safety of SGLT2 inhibitors for HFpEF in order to provide evidence for clinical application.

## Methods

The review protocol was registered with PROSPERO (No: CRD42021291122).^1^ This study was carried out according to the Cochrane Handbook for Systematic Reviews of Interventions (31) and was reported according to the Preferred Reporting Items for Systematic reviews and Meta-Analyses (PRISMA) (32).

### Literature Search

The literature searches were conducted in the following eight databases: MEDLINE, EMBASE, Ovid, Cochrane Library, Chinese National Knowledge Infrastructure Database, VIP information database, Chinese Biomedical Database, and Wanfang Data Information Site. The publication time was set from the inception to November 14, 2021. We used the following MeSH terms in conjunction with free-text terms to perform search: heart failure, sodium-glucose cotransporter 2 inhibitors, SGLT2 inhibitors, ertugliflozin, canagliflozin, dapagliflozin, empagliflozin, ipragliflozin, tofogliflozin, and luseogliflozin. Bibliographies of the retrieved articles were searched for potential eligible studies. We returned to search just before the final analyses and further studies retrieved for inclusion.

### Eligibility Criteria

Original literature was included if it met the following inclusion criteria: (1) Types of studies (S): randomized controlled trials (RCTs); (2) Types of participants (P): in patients with HFpEF or in a subgroup of patients with HFpEF within the trial; (3) Types of interventions (I): SGLT2 inhibitors; (4) Types of comparators (C): placebo, no drug or antidiabetics; (5) Types of outcome measures (O): reporting at least one of the clinical outcomes of interest (including cardiovascular events, echocardiographic measures, adverse events and so on). Exclusion criteria: (1) duplicate publications; (2) trials whose allocation methods use date of birth, date of admission, hospital numbers, or alternation; (3) overlapping patient populations; (4) adopted crossover design. RCTs of SGLT2 inhibitors in patients with and without HFpEF were eligible only when they reported specific outcomes in the HFpEF population.

### Data Extraction

Data extraction was performed independently by two reviewers (Zhou HF, Lin Q). The extracted data included: authors, title of study, year of publication, sample size, treatment duration, hazard ratios and confidence intervals for the outcomes of interest and other PICOS details. We defined a composite of first hospitalization for heart failure (HHF) or cardiovascular (CV) death as the primary outcome. Secondary endpoints were first hospitalization for heart failure, cardiovascular death, all-cause mortality, total hospitalization for heart failure, the ratio of early mitral inflow velocity to mitral annular early diastolic velocity (E/e’), N-terminal pro-B-type natriuretic peptide (NT-proBNP), B-type natriuretic peptide (BNP), and 6-min walk test distance (6MWTD). Adverse events (AEs) were defined as any unfavorable or unintended sign, symptom, or disease, including abnormal laboratory values.

### Risk of Bias Assessment

According to the Cochrane Handbook for Systematic Reviewers of Interventions version 5.1.0 (31), two reviewers (Zhou HF, Lin Q) independently assessed the risk of bias for each included study. The items of risk of bias were consist of random sequence generation (selection bias), allocation concealment (selection bias), participant and personnel blinding (performance bias), outcome assessment blinding (detection bias), incomplete outcome data (attrition bias), selective reporting (reporting bias), and baseline data comparability (other bias). Each item was given a risk of bias rating of low, uncertain, or high. Disagreements were settled through discussion, with the involvement of a third review author (Wang X) when necessary. In addition, we evaluated the quality of included evidences using the GRADE (grading of recommendations assessment, development, and evaluation) method (33).

### Data Analysis

The primary outcome, first HHF and CV death were compared using pooled hazard ratios (HR) and 95% confidence intervals (CI) to preserve time-to-event data from individual studies. Risk ratio (RR) was used to pool other binary endpoints and mean differences (MD) were used to pool continuous outcomes. If a continuous outcome was expressed in the interquartile range, we performed the analysis using metabin, metacount and metareg functions of the meta library of R 3.5.1 (R Foundation for Statistical Computing, Vienna, Austria)^2^ (34, 35). If a continuous outcome was expressed in mean and standard deviation, we conducted the analyses using Review Manager 5.1 (Nordic Cochrane center, The Cochrane Collaboration, Copenhagen, Denmark). The I-squared statistic and Cochrane’s Q test were used to analyze between-trial heterogeneity. According to the Cochrane Handbook for Systematic Reviews of Interventions, the *I*^2^ scale ranged from 0 to 100%, with values ranging from 0 to 40%, 30 to 60%, 50 to 90%, and 75 to 100% indicating that heterogeneity might not be important, moderate heterogeneity, substantial heterogeneity, and considerable heterogeneity, respectively (31, 36). We used a random-effects model to evaluate the overall effect in heterogeneous studies because random-effects models assess the study’s outcomes based on within-trial and between-trial variance (37), which providing more cautious conclusions. The sensitivity analysis was also performed by removing each study one at a time to evaluate the stability of the results. Because the results may be influenced by differences in the left ventricular ejection fraction thresholds used in the diagnosis of HFpEF, subgroup analysis was performed according to various thresholds of the left ventricular ejection fraction (40, 45, or 50%). The publication bias was detected by the funnel plot, the Begger’s test and the Egger’s test (38).

## Results

### Study Selection

**FIGURE 1:**
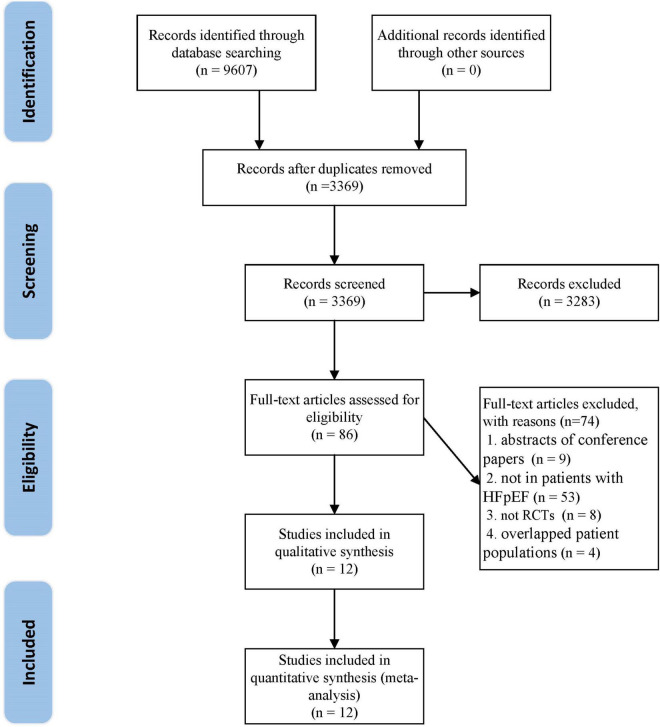
The flow chart of the study selection process showing how to screen eligible randomized controlled trials.

A total of 9,607 articles were retrieved from the initial search. After deleting the duplicate literature, 3,369 articles remained. After reading the title and abstract, 3,283 articles were excluded, and 86 articles were screened in detail. By reading the full text of the remaining 86 articles, 74 articles were excluded (Supplementary Table 1), which did not meet our inclusion criteria: eight were not RCTs; nine were abstracts of conference papers; 53 were not in patients with HFpEF; four were overlapped patient populations. Finally, 12 trials (29, 30, 39–48) were qualified and included in the meta-analysis. The flow chart of literature screening is as follows (Figure 1).

### Study Characteristics

A total of 10,883 patients with HFpEF from 12 RCTs were included in this systematic review and meta-analysis, of which 5,621 patients were assigned to the SGLT2 inhibitors group and 5,262 patients were assigned to the control group. The median follow-up period ranged from 3 to 50.4 months. Five studies were large-scale clinical trials that were carried out only in patients with HFpEF: EMPERIAL- Preserved (39), EMPEROR-Preserved (30), MUSCAT-HF (40), PRESERVED-HF (41), and CANONICAL (42). Five studies were *post hoc* and subgroup analyses of large-scale cardiovascular outcome trials: SCORED (43), DECLARE-TIMI 58 (44), SOLOIST-WHF (45), VERTIS CV (29), and CANDLE (46). In addition, we included two smaller studies, which were conducted in patients with HFpEF and conduct in Russia (47) and China (48), respectively. Dapagliflozin (10 mg per day) was used in three RCTs (41, 44, 48), empagliflozin (10 mg per day) was used in three RCTs (30, 39, 47), canagliflozin (100 mg per day) was used in two RCTs (42, 46), sotagliflozin (200 mg per day) was used in two RCTs (43, 45), and ertugliflozin (5 mg per day) (29) and luseogliflozin (2.5 mg per day) (40) were used in one RCT each. Seven studies were placebo-controlled trials (29, 30, 39, 41, 43–45), and the other five trials compared SGLT2 inhibitors with antidiabetics. Five RCTs provided data for a composite of first hospitalization for heart failure or cardiovascular death (29, 30, 43–45), three RCTs provided data for first hospitalization for heart failure (29, 30, 44), three RCTs provided data for cardiovascular death (29, 30, 44), five RCTs provided data for all-cause mortality (29, 30, 41, 44, 46), and four RCTs provided data for total hospitalization for heart failure (30, 42, 44, 46). The data of 6MWTD, BNP and E/e’ were all changes from baseline to the end of the treatment. 6MWTD was reported in two RCTs (39, 47), BNP was in 2 RCTs (41, 42), and E/e’ was in 2 RCTs (46, 48). The changes of NT-proBNP from baseline to the end of the treatment were reported in 3 trials (30, 40, 47), while the NT-proBNP at the end of the treatment were reported in other 3 trials (41, 46, 48). Of the 12 trials, 7 trials reported a total of 3,290 AEs (30, 39–42, 47, 48). Two studies using a left ventricular ejection fraction > 40% as a cut-off point for HFpEF (30, 39), and four studies using a left ventricular ejection fraction > 45% as a cut-off point for HFpEF (29, 40, 41, 44), while the other trials used a threshold of left ventricular ejection fraction > 50% (42, 43, 45–48). Characteristics of the trials and the detail of PICOS are shown in Table 1.

**TABLE 1:** Characteristics of the included RCTs and the detail of PICOS.

Include studies	Participants	Sample size (T/C)	Intervention		Therapeutic course	Outcomes	Diagnostic thresholds of LVEF	Types of studies
			T	C				
EMPERIAL-Preserved 2021	HFpEF	157/158	Empagliflozin 10 mg qd	Placebo	12 weeks	⑥	LVEF > 40%	HFpEF-specific trials
EMPEROR-Preserved 2021	HFpEF	2,997/2,991	Empagliflozin 10 mg qd	Placebo	26.2 months	①②③④⑤⑦	LVEF > 40%	HFpEF-specific trials
SOLOIST-WHF 2021	HFpEF	127/129	Sotagliflozin 200 mg qd	Placebo	9.2 months	①	LVEF > 50%	*Post hoc* studies
VERTIS CV 2020	HFpEF	690/327	Ertugliflozin 5 mg qd	Placebo	3.5 years	①②③⑤	LVEF > 45%	*Post hoc* studies
MUSCAT-HF 2020	HFpEF	83/82	Luseogliflozin 2.5 mg qd	Voglibose 0.2 mg tid	12 weeks	⑦⑨	LVEF > 45%	HFpEF-specific trials
PRESERVED-HF 2021	HFpEF	162/162	Dapagliflozin 10 mg qd	Placebo	12 weeks	⑤⑦⑧	LVEF > 45%	HFpEF-specific trials
DECLARE-TIMI 58 2019	HFpEF	399/409	Dapagliflozin 10 mg qd	Placebo	4.2 years	①	LVEF > 45%	*Post hoc* studies
CANDLE 2020	HFpEF	78/87	Canagliflozin 100 mg qd	Glimepiride 0.5 mg qd	24 weeks	④⑤⑦⑨	LVEF > 50%	*Post hoc* studies
(Continued on next page)								
CANONICAL 2021	HFpEF	42/40	Canagliflozin 100 mg qd	Standard diabetic therapy	24 weeks	④⑧	LVEF > 50%	HFpEF-specific trials
Borisov 2021	HFpEF	30/30	Empagliflozin 10 mg qd	Standard diabetic therapy	24 weeks	⑥⑦⑨	LVEF > 50%	HFpEF-specific trials
Sun 2021	HFpEF	23/23	Dapagliflozin 10 mg qd	Standard diabetic therapy	24 weeks	⑦⑨	LVEF > 50%	HFpEF-specific trials
SCORED 2021	HFpEF	843/824	Sotagliflozin 200 mg qd	Placebo	16 months	①	LVEF > 50%	*Post hoc* studies

*T, treatment; C, control; HFpEF, Heart failure with preserved ejection fraction; LVEF, left ventricular ejection fraction;①, a composite of first hospitalization for heart failure or cardiovascular death; ②, time to first hospitalization for heart failure; ③, cardiovascular death; ④, total number of hospitalization for heart failure; ⑤, death from any cause; ⑥, 6-min walk test distance; ⑦, N-terminal pro-B-type natriuretic peptide; ⑧, B-type natriuretic peptide; ⑨, the ratio of early mitral inflow velocity to mitral annular early diastolic velocity.*

### Risk of Bias in Individual Studies

**FIGURE 2:**
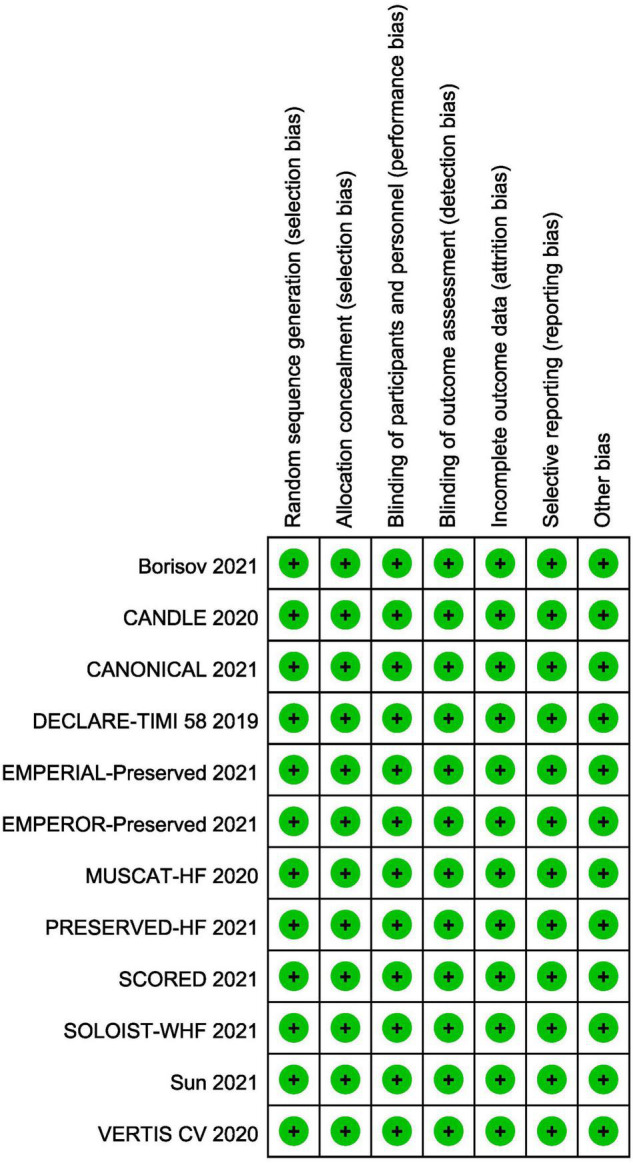
Risk of bias summary.

All included trials were at low risk of bias. Randomization, allocation concealment and blind method had been mentioned and explicitly described in all studies. The summary of risk of bias is shown in Figure 2. Supplementary Table 2 summarized the level of evidence for the studies included and indicated that the overall quality of the evidence was high.

### Overall Results of Meta-Analysis

#### Composite of First Hospitalization for Heart Failure or Cardiovascular Death

**FIGURE 3:**
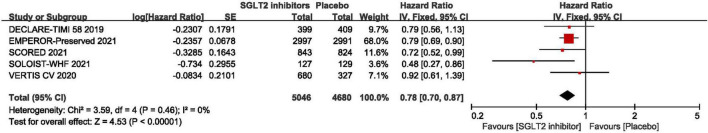
Forest plot displaying the effects of SGLT2 inhibitors vs. placebo for composite of cardiovascular death or first hospitalization arises from heart failure in HFpEF patients.

**FIGURE 4:**
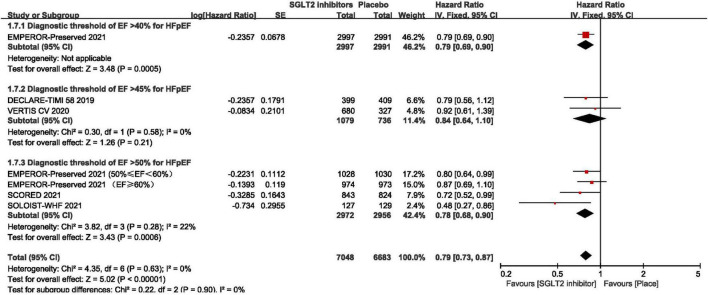
Forest plot displaying the results of subgroup analysis by various thresholds of ejection fraction used in the diagnosis of HFpEF.

**FIGURE 5:**
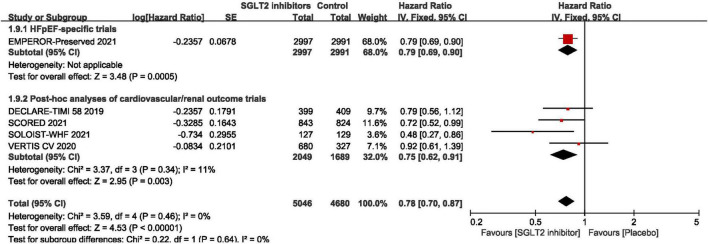
Forest plot displaying the results of subgroup analysis by types of studies.

As shown in Figure 3, pooled result of the 5 trials (29, 30, 43–45) revealed that SGLT2 inhibitors significantly reduced the composite of first hospitalization for heart failure or cardiovascular death compared to placebo in patients with HFpEF (HR:0.78, 95% CI: [0.70, 0.87], *P*<0.00001, *I*^2^ = 0%). According to various thresholds of left ventricular ejection fraction (40, 45, or 50%) used in the diagnosis of HFpEF, the subgroup analysis revealed that when using a left ventricular ejection fraction > 50% as the cut-off point for HFpEF, SGLT2 inhibitors also significantly reduced the composite endpoint of the first hospitalization for heart failure or cardiovascular death in patients with HFpEF (HR: 0.78, 95% CI: [0.68, 0.90], *P* = 0.0006, *I*^2^ = 22%) (as shown in Figure 4). In the 5 trials, one trials was HFpEF-specific trial and four were *post hoc* analyses of cardiovascular/renal outcome studies. The findings of the HFpEF-specific trials (HR: 0.79, 95% CI: [0.69, 0.90], *P* = 0.0005) and the *post hoc* studies (HR: 0.75, 95% CI: [0.62, 0.91], *P* = 0.003, *I*^2^ = 11%) were consistent (as shown in Figure 5).

#### First Hospitalization for Heart Failure

**FIGURE 6:**
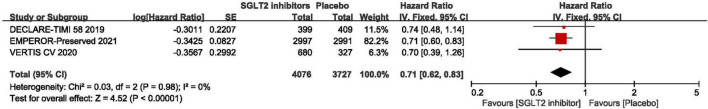
Forest plot displaying the effects of SGLT2 inhibitors vs. placebo for first hospitalization for heart failure in HFpEF patients.

The result of the Meta-analysis of three studies (29, 30, 44) demonstrated that SGLT2 inhibitors significantly reduced the number of first hospitalization for heart failure in patients with HFpEF compared to placebo (HR: 0.71, 95% CI: [0.62, 0.83], *P* < 0.00001, *I*^2^ = 0%). The result is shown in Figure 6.

#### Cardiovascular Death

**FIGURE 7:**
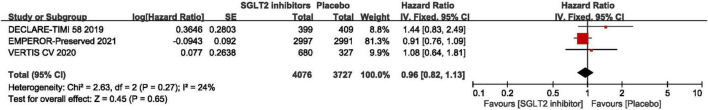
Forest plot displaying the effects of SGLT2 inhibitors vs. placebo for cardiovascular death in HFpEF patients.

As shown in Figure 7, the pooled result of three RCTs (29, 30, 44) revealed that there was no significant difference between SGLT2 inhibitors and placebo in terms of cardiovascular death (HR: 0.96, 95% CI: [0.82, 1.13], *P* = 0.65, *I*^2^ = 24%).

#### Total Hospitalization for Heart Failure

**FIGURE 8:**
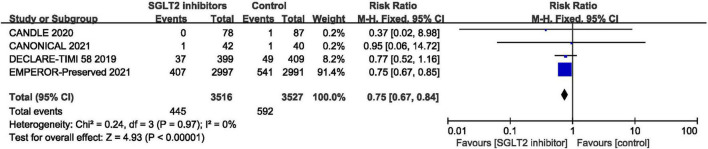
Forest plot displaying the effects of SGLT2 inhibitors vs. placebo for total hospitalization for heart failure in HFpEF patients.

In terms of the total hospitalization for heart failure, which included first and recurrent number of hospitalization for heart failure, the result of meta-analysis of four studies (30, 42, 44, 46) demonstrated that SGLT2 inhibitors significantly reduced the total hospitalization for heart failure in patients with HFpEF compared to placebo (RR:0.75, 95% CI: [0.67, 0.84], *P*<0.00001, *I*^2^ = 0%). The result is shown in Figure 8.

#### All-Cause Mortality

**FIGURE 9:**
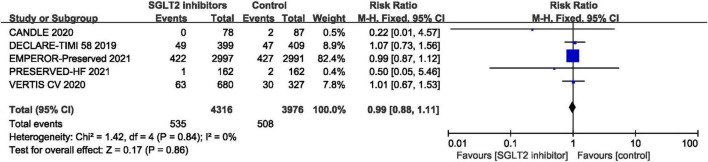
Forest plot displaying the effects of SGLT2 inhibitors vs. placebo for all-cause mortality in HFpEF patients.

Five RCTs provided data for all-cause mortality (29, 30, 41, 44, 46) and pooled result shown that no statistical difference was found between SGLT2 inhibitors and placebo in terms of the all-cause mortality (RR: 0.99, 95% CI: [0.88, 1.11], *P* = 0.86, *I*^2^ = 0%). The result is shown in Figure 9.

#### E/e’

**FIGURE 10:**
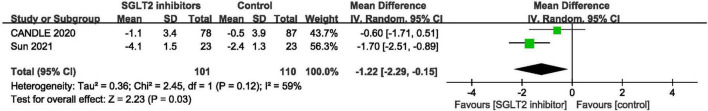
Forest plot displaying the effects of SGLT2 inhibitors vs. placebo for E/e’ in HFpEF patients.

The ratio of early mitral inflow velocity to mitral annular early diastolic velocity was expressed in mean and standard deviation in two trials (46, 48). Pooled result demonstrated that SGLT2 inhibitors significantly reduced the E/e’ in patients with HFpEF compared to control (MD: –1.22, 95% CI: [–2.29, –0.15], *P* = 0.03, *I*^2^ = 59%). The result is shown in Figure 10.

#### N-Terminal Pro-B-Type Natriuretic Peptide

The changes of NT-proBNP from baseline to the end of the treatment were reported in 3 trials (30, 40, 47), while the NT-proBNP at the end of the treatment were reported in other 3 trials (41, 46, 48). The results of these two forms were pooled separately and both showed that no difference were found between groups (MD: –26.60, 95% CI: [–61.20, 7.99], *P* = 0.13, *I*^2^ = 98%; MD: –8.51, 95% CI: [–33.19, 16.16], *P* = 0.50, *I*^2^ = 0%; respectively) (Supplementary Table 3).

#### B-Type Natriuretic Peptide

In terms of BNP, the pooled result of the two trials (41, 42) revealed that no statistical difference was found between groups (MD: –21.04, 95% CI: [–75.69, 33.62], *P* = 0.45, *I*^2^ = 72%) (Supplementary Table 3).

#### 6-Min Walk Test Distance

Meta-analysis of 2 studies (39, 47) showed that no statistically significant difference was found between groups in terms of 6MWTD (MD: 14.99, 95% CI: [–4.60, 34.60], *P* = 0.13, *I*^2^ = 87%) (Supplementary Table 3).

#### Adverse Events

**FIGURE 11:**
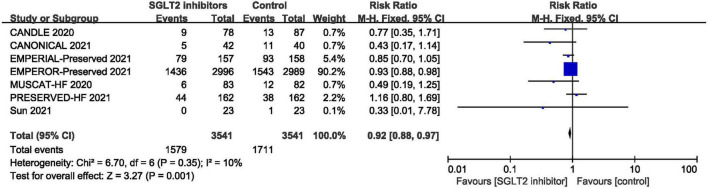
Forest plot displaying the effects of SGLT2 inhibitors vs. placebo for adverse events in HFpEF patients.

Pooled result of the seven trials (30, 39–42, 47, 48) revealed that the incidence of adverse events in SGLT2 inhibitors group was significantly lower than that in control group (RR: 0.92, 95% CI: [0.88, 0.97], *P* = 0.001, *I*^2^ = 0%). The result is shown in Figure 11.

### Sensitivity Analysis

Sensitivity analysis was performed by removing trials from the analysis one at a time to see how they affected the results. The results showed that when a single trial was excluded from the analysis, the cumulative effects of SGLT2 inhibitors on HFpEF did not vary significantly (Supplementary Figure 1).

### Publication Bias

**FIGURE 12:**
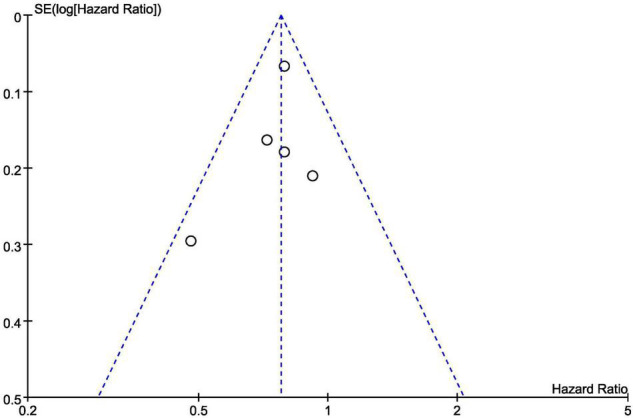
Funnel plot of the included trials with composite of cardiovascular death or first hospitalization for heart failure data.

Figure 12 is a funnel diagram of the impact of SGLT2 inhibitors on heart failure with preserved ejection fraction, suggesting that there was no evidence of publication bias. The results of the Begg’s and Egger’s tests were z = 0.24 (*P* = 0.806) and *t* = –0.82 (*P* = 0.473), respectively, showing that there was also no publication bias in statistics.

## Discussion

In this meta-analysis, 12 RCTs with a total of 10,883 patients were included to assess the effect of SGLT2 inhibitors in patients with HFpEF. It showed that SGLT2 inhibitors could significantly reduce the composite of first hospitalization for heart failure or cardiovascular death, total hospitalization for heart failure, and first hospitalization for heart failure in patients with HFpEF. However, no statistical differences were found between SGLT2 inhibitors and placebo in terms of cardiovascular death or all-cause mortality. In addition, SGLT2 inhibitors could improve ventricular diastolic function by lower the ratio of early mitral inflow velocity to mitral annular early diastolic velocity, but no improvements were found in NT-proBNP, BNP and 6MWTD. In terms of AEs, the SGLT2 inhibitors could reduce the incidence of adverse events. SGLT2 inhibitors may be safe and effective in the treatment of HFpEF and have great potential as a new option for HFpEF therapy. However, these findings should be considered exploratory rather than definitive due to the availability of scarce data. There is currently a large RCT underway, the DELIVER trial (Dapagliflozin Evaluation to Improve the Lives of Patients with Preserved Ejection Fraction Heart Failure; NCT03619213), which may provide further evidence of the efficacy of SGLT2 inhibitors in the treatment of HFpEF.

In this meta-analysis, SGLT2 inhibitors were not found to be superior to placebo in terms of cardiovascular death and all-cause mortality, which is similar to the findings of another potential treatment for HFpEF called Sacubitril/Valsartan (16, 49). Neither SGLT2 inhibitors nor Sacubitril/Valsartan could significantly reduce the all-cause mortality and cardiovascular mortality in patients with HFpEF. The reasons for these results are not yet clear. Current studies have found that both SGLT2 inhibitors and Sacubitril/Valsartan can inhibit the renin-angiotensin-aldosterone system (RAAS), causing dilation of afferent glomerular arteriole and efferent arteriole, resulting in a decrease in renal arterial pressure, causing a decrease in renal perfusion and thus a decrease in glomerular filtration rate (GFR) and ultimately in renal function (50–52). This effect is generally considered deleterious, as data from large epidemiological studies and meta-analyses suggest that even a slight decrease in eGFR is associated with an increased risk of adverse clinical outcomes (53–55). In HFpEF, inhibition of the RAAS system is associated with a 50% increased risk of renal dysfunction (52). Decreased renal function can lead to increased mortality, which counteracts the positive effect of SGLT2 inhibitors and Sacubitril/Valsartan in reducing cardiovascular mortality or all-cause mortality, and may result in no significant difference in reducing cardiovascular or all-cause death compared with placebo. On the other hand, the EMPEROR-Preserved study showed a high proportion of patients discontinuing treatment for reasons other than death, which may have tipped the effect size toward the null hypothesis (30). In this meta-analysis, no significant improvements in NT-proBNP, BNP, and 6MWTD were found, which may be related to the following factors. First, the majority of patients who were enrolled in the included studies had low levels of NT-proBNP, BNP, and NYHA classification, which may diminish the effect of SGLT2 inhibitors in this study. Furthermore, these results may be related to the shorter follow-up period of the included studies. Several recent studies in HFrEF patients also found that the application of SGLT2 inhibitors treatment for 12 weeks improved the clinical outcomes, but did not affect the level of NT-proBNP (39). These conflicting findings should be interpreted as suggesting that there may be a disconnect between short-term changes in NT-proBNP levels and clinical outcomes (25, 46, 56). Moreover, studies have shown that cardiac performance was not related to exercise capacity (39, 56, 57); therefore, it was not unexpected that this meta-analysis did not show an improvement in the endpoint of exercise capacity as measured by the 6MWTD (39).

The mechanisms by which SGLT2 inhibitors improve cardiovascular prognosis remain less clear. Diuretic and natriuretic effects may play an important role in the treatment of HFpEF by SGLT2 inhibitors (58), which is similar to that seen with SGLT2 inhibitors in patients with HFrEF (59). Because the resorption of glucose and sodium in the proximal convoluted tubule is coupled (60), SGLT2 inhibitors can cause natriuresis by inhibiting the transport of sodium for every molecule of unabsorbed glucose, leading to the reduction in plasma volume and blood pressure, which improves cardiac afterload (61, 62). However, some studies have also found that the natriuretic effect is typically mild and short-lived due to activation of systemic renin-angiotensin-aldosterone as a compensatory mechanism, leading to subsequent recovery of urine output (58, 63–65). Excessive sympathetic nerve activity plays an important role in the progression of HFpEF (66). Diastolic dysfunction is a characteristic manifestation of HFpEF (67), and the more severe the diastolic dysfunction is, the worse the prognosis of HFpEF becomes (68). Several studies have found that sympathetic nervous overactivity may lead to the development of diastolic dysfunction (69). Impaired myocardial sympathetic innervation, which reflects sympathetic overactivity, is associated with the severity of diastolic dysfunction in patients with HFpEF (70). Preclinical studies have shown that elevated sympathetic activity simulated by isoproterenol administration leads to diastolic dysfunction, with myocardial stiffness, fibrosis, and left ventricular hypertrophy (71). Excessive sympathetic stimulation causes desensitization and downregulation of β-adrenergic receptors, leading to cardiac remodeling as well as worsening of HFpEF (72). It was found that the SGLT2 inhibitors could improve diastolic function by reducing sympathetic tone (73). Inflammation and oxidative stress have been implicated in the pathogenesis of HFpEF (74, 75). Oxidative stress and inflammation can lead to expanded epicardial adipose tissue mass, microvascular endothelial dysfunction, increased arterial wall stiffness, and fibrosis of the underlying myocardium, normal to mildly increased left ventricular volumes and systolic blood pressures, which can result in HFpEF (76, 77). Studies have shown that SGLT2 inhibitors could reduce inflammatory reaction and oxidative stress in HFpEF, thereby improving microcirculatory dysfunction, reducing vascular stiffness, and systemic blood pressure (78, 79). Furthermore, SGLT2 inhibitors may reduce epicardial adipose tissue, which could ultimately lead to improved distensibility (80). Several studies have also shown that SGLT2 inhibitors could reduce excessive diastolic tension and decrease LV mass, improving cardiac preload (81, 82). Moreover, SGLT2 inhibitors may ameliorate symptoms of HFpEF in part due to their interference with metabolic pathways (83). SGLT2 inhibitors induce ketogenic metabolism, which results in utilization of energy-efficient ketones over less efficient fatty acid and glucose oxidation to generate myocardial energy, thereby improving efficiency and function of both myocardium and the kidneys (81, 84, 85). Additional mechanisms of SGLT2 inhibitors that might be beneficial include increased hematocrit level (86), inhibition of the Na^+^/H^+^-exchanger (87), prevention of adverse cardiac remodeling (88), prevention of ischemia/reperfusion injury (89), reduced serum uric acid level (90), reduced glomerular hyperfiltration and albuminuria (51), and inhibition of the sympathetic nervous system (50). Whether the efficacy of SGLT2 inhibitors in the treatment of heart failure with preserved ejection fraction can be explained by these mechanisms remains to be fully explored.

In terms of the risk of bias, the main risk of bias may lie in the definition of HFpEF varied across trials. Through the subgroup analysis we found that SGLT2 inhibitors significantly reduced the composite endpoint of the first hospitalization for heart failure or cardiovascular death in patients with HFpEF no matter using a left ventricular ejection fraction > 50%, or > 40% as the cut-off point for HFpEF. However, two trials revealed that SGLT2 inhibitors were not superior to control when using a left ventricular ejection fraction > 45% as a cut-off point for HFpEF. Due to these differences, a sensitivity analysis by removing the studies corresponding to the same diagnostic criteria separately was conducted, and the results were stable. Although sensitivity analysis showed stable results, considering the differences between different diagnostic criteria, it may lead to selection bias by improper selection of subjects making the study results deviate from the true picture.

### Comparison With Previous Studies

Previous meta-analyses have focused mainly on the effects of SGLT2 inhibitors on heart failure or HFrEF, but there was no HFpEF-specific meta-analysis, or only described that in the subgroup analysis. Also, the only outcome studied for the subgroup analyses was the composite of cardiovascular death and hospitalizations for heart failure, but the individual endpoints of all-cause mortality, cardiovascular mortality, and hospitalization for heart failure in HFpEF were not analyzed. Our meta-analysis has several advantages over previous meta-analyses. First, we included several recently published and well-conducted trials in our meta-analysis. Second, in the meta-analysis, we performed subgroup analyses on different aspects, which made the results more stable.

The result of our meta-analysis is similar to that of previous meta-analyses regarding the composite of cardiovascular death and HF hospitalizations. Additionally, we newly found that SGLT2 inhibitors significantly reduced hospitalization for heart failure and improved ventricular diastolic function as measured by the ratio of early mitral inflow velocity to mitral annular early diastolic velocity. Furthermore, regardless of the threshold of left ventricular ejection fraction (40 or 50%) used in the diagnosis of HFpEF in included trials, SGLT2 inhibitors could improve the cardiovascular outcomes in patients with HFpEF, further strengthening the efficacy of SGLT2 inhibitors.

### Limitation

There are several limitations to this meta-analysis. First, not all studies have published the necessary subgroup data for all endpoints. Therefore, some of these studies were not included in the analysis of individual endpoints. Second, we pooled the outcomes of all SGLT2 inhibitors under the same intervention group, and did not perform a subgroup analysis by drug categories due to the small number of included studies. Whether there are differences in outcomes between different drugs could not be assessed in our study. Furthermore, the definition of HFpEF varies from trial to trial, and it may lead to selection bias due to different subject selection, skewing the study results from the true picture. Moreover, five of the included studies were *post hoc* subgroup analyses of large-scale studies and did not provide detailed descriptions of baseline patient-level characteristics for the subgroup of HFpEF, so it was not possible to perform subgroup analyses by age, sex, race, renal function, and presence of diabetes, and therefore possible differences in outcomes arising from these subgroup factors could not be assessed. Additionally, in the *post hoc* subgroup analyses, differences in baseline patient-level characteristics between the SGLT2 inhibitors and placebo groups may limit interpretation. Also, the pooled number of events could not be reported due to the lack of patient-level data. Instead, we calculated and reported hazard ratios. In addition, there were differences in study design, subject characteristics, sample size, and follow-up time among the 12 RCTS, which may result in selection bias and measurement bias, leading to inaccurate results. Finally, we admit that using funnel plots to identify publication bias is less reliable when the meta-analysis contains less than 10 trials in total.

### Implications for Research

The systematic review and meta-analysis provide a certain amount of evidence for SGLT2 inhibitors in improving the prognosis of patients with HFpEF. The reduction in hospitalization endpoints indicates that SGLT2 inhibitors should be considered as part of standard care in patients with HFpEF. For future studies, the definition of HFpEF should use a threshold of left ventricular ejection fraction > 50%. Furthermore, outcome measures should include not only the number of cardiovascular events, but also parameters of ventricular diastolic function assessed by echocardiography to evaluate the efficacy of SGLT2 on HFpEF more comprehensively. Moreover, since HFpEF may fluctuate in a long course, continuous follow-up is important to determine the true efficacy and long-term effect of SGLT2 inhibitors. In addition, existing studies have not found that SGLT2 inhibitors can significantly reduce cardiovascular mortality in patients with HFpEF. Therefore, exploring the effect of SGLT2 inhibitors on cardiovascular mortality in patients with HFpEF is another problem we will face in the future. Finally, in future studies, it should be assessed whether there are differences in cardiovascular prognosis between different SGLT2 inhibitors.

## Conclusion

SGLT2 inhibitors significantly improve cardiovascular outcomes including hospitalization for heart failure and ventricular diastolic function with a decreased risk of serious adverse events in patients with HFpEF. However, due to the limited number of RCTs available at this time, these findings require careful recommendation. There is a need for more multi-center, randomized, double-blind, placebo-controlled studies that meet the CONSORT 2010 guidelines.

## Data Availability Statement

The original contributions presented in the study are included in the article/Supplementary Material, further inquiries can be directed to the corresponding author/s.

## Author Contributions

HZ and YZ conceived, drafted this systematic review, and registered the protocol at PROSPERO. HZ and QL developed the search strategy and conducted the literature research, study selection, data extraction, and risk of raise assessment. YW, BW, YD, GP, and WP interpreted the evidence from methodological and clinical perspective. HZ and FL contributed to the drafting of manuscript. XW oversaw the conduct of the study. All authors have read, critically reviewed, and approved the final manuscript.

## Conflict of Interest

The authors declare that the research was conducted in the absence of any commercial or financial relationships that could be construed as a potential conflict of interest.

## Publisher’s Note

All claims expressed in this article are solely those of the authors and do not necessarily represent those of their affiliated organizations, or those of the publisher, the editors and the reviewers. Any product that may be evaluated in this article, or claim that may be made by its manufacturer, is not guaranteed or endorsed by the publisher.

## Footnotes

1
https://www.crd.york.ac.Uk/prospero/

2
http://www.r-project.org
