# Supplementary material for: Effect of Sodium-Glucose Cotransporter 2 Inhibitors for Heart Failure With Preserved Ejection Fraction: A Systematic Review and Meta-Analysis of Randomized Clinical Trials
Source: Front Cardiovasc Med. 2022 May 4;9:875327. doi: 10.3389/fcvm.2022.875327 (PMC9116195; doi:10.3389/fcvm.2022.875327)

Supplementary Online Content

Hufang Zhou, Wenhua Peng, Fuyao Li, Yuelin Wang, Baofu Wang, Yukun Ding, Qian Lin, Ying Zhao, Guozhong Pan, Xian Wang. Effect of Sodium-Glucose Cotransporter 2 Inhibitors for Heart Failure with Preserved Ejection Fraction: A Systematic Review and Meta-analysis of Randomized Clinical Trials

eMethods. Search Strategies

eTable 1. List of studies excluded at full-text screening stage

eTable 2. GRADE summary of evidence for effect of SGLT2 inhibitors on heart failure with preserved ejection fraction

eTable 3. Effect of SGLT2 inhibitors compared with control on NT-proBNP, BNP and 6MWTD in patients with heart failure with preserved ejection fraction

eFigure 1. Sensitivity analysis of the composite endpoint in patients with HFpEF treated by SGLT2 inhibitors

e Methods. Search Strategies

(1) **Search strategy for Medline Database (November 14, 2021)**

#1 heart failure [mh]

#2 heart failure [tiab]

#3 #1 OR #2

#4 Sodium-Glucose Transporter 2 Inhibitors [mh]

#5 Sodium-Glucose Transporter 2 Inhibitors [tiab] OR sodium-glucose cotransporter 2 inhibitors [tiab] OR SGLT2 [tiab] OR Ertugliflozin [tiab] OR Canagliflozin [tiab] OR Dapagliflozin [tiab] OR Empagliflozin [tiab] OR Ipragliflozin [tiab] OR Tofogliflozin [tiab] OR Luseogliflozin [tiab]

#6 #4 OR #5

#7 #3 AND #6

Find literatures：1673

(2) **Search strategy for Embase (November 14, 2021)**

#1 'sodium glucose cotransporter 2 inhibitor':ab,ti OR 'sodium-glucose cotransporter 2 inhibitors':ab,ti OR sglt2:ab,ti OR ertugliflozin:ab,ti OR canagliflozin:ab,ti OR dapagliflozin:ab,ti OR empagliflozin:ab,ti OR tofogliflozin:ab,ti OR luseogliflozin:ab,ti OR ipragliflozin:ab,ti

#2 'heart failure':ab,ti

#3 #1 AND #2

Find literatures：2190

(3) **Search strategy for Ovid database (November 14, 2021)**

#1 (Sodium-Glucose Transporter 2 Inhibitors or sodium-glucose cotransporter 2 inhibitors or SGLT2 or Ertugliflozin or Luseogliflozin or Canagliflozin or Tofogliflozin or Ipragliflozin or Empagliflozin or Dapagliflozin).ab.

#2 heart failure.ab.

#3 1 and 2

Find literatures：3723

(4) **Search strategy for Cochrane Library (November 14, 2021)**

#1 (Sodium-Glucose Transporter 2 Inhibitors):ti,ab,kw
#2 (sodium-glucose cotransporter 2 inhibitors):ti,ab,kw
#3 (SGLT2):ti,ab,kw
#4 (Ertugliflozin):ti,ab,kw
#5 (Canagliflozin):ti,ab,kw
#6 (Dapagliflozin):ti,ab,kw
#7 (Empagliflozin):ti,ab,kw
#8 (Tofogliflozin):ti,ab,kw
#9 (Luseogliflozin):ti,ab,kw
#10 (Ipragliflozin):ti,ab,kw
#11 #1 or #2 or #3 or #4 or #5 or #6 or #7 or #8 or #9 or #10
#12 (heart failure):ti,ab,kw
#13 #11 and #12

Find literatures：763

（5）Search strategy for Chinese National Knowledge Infrastructure Database (November 14, 2021)

SU=('sodium-glucose cotransporter 2 inhibitors'+'SGLT2'+'Ertugliflozin'+'Canagliflozin'+'Dapagliflozin'+'Empagliflozin'+'Ipragliflozin'+'Tofogliflozin'+'Luseogliflozin')*'heart failure'

Find literatures：1036

(6) Search strategy for Wanfang Data Information Site (November 14, 2021)

theme:("sodium-glucose cotransporter 2 inhibitors" or "SGLT2" or "Ertugliflozin" or "Canagliflozin" or "Dapagliflozin" or "Empagliflozin" or "Ipragliflozin" or "Tofogliflozin" or "Luseogliflozin") and theme:("heart failure")

Find literatures：63

(7) Search strategy for Chinese Biomedical Database (November 14, 2021)

#1 "sodium-glucose cotransporter 2 inhibitors"[abstract] OR "SGLT2"[abstract] OR "Ertugliflozin"[abstract] OR "Canagliflozin"[abstract] OR "Dapagliflozin"[abstract] OR "Empagliflozin"[abstract] OR "Ipragliflozin"[abstract] OR "Tofogliflozin"[abstract] OR "Luseogliflozin"[abstract]

#2 "heart failure"[abstract]

#3 (#1) AND (#2)

Find literatures：26

(8) Search strategy for VIP information database (November 14, 2021)

M=(sodium-glucose cotransporter 2 inhibitors + SGLT2 + Ertugliflozin + Canagliflozin + Dapagliflozin + Empagliflozin + Ipragliflozin + Tofogliflozin + Luseogliflozin) * R=(heart failure) * R=(preserved)

Find literatures：133

e Table 1. List of studies excluded at full-text screening stage

Number	Author	Title	Reason
1	Nassif, M.	Empagliflozin Effects on Pulmonary Artery Pressure in Patients With Heart Failure: Results From the EMBRACE-HF Trial	not in patients with HFpEF
2	Heerspink, H.J.L.	Effects of dapagliflozin on mortality in patients with chronic kidney disease: a pre-specified analysis from the DAPA-CKD randomized controlled trial	not in patients with HFpEF
3	Inzucchi, S.E.	Empagliflozin reduces the total burden of all-cause hospitalisations and all-cause mortality in the EMPA-REG OUTCOME trial	not in patients with HFpEF
4	McMurray, J.	Effects of Dapagliflozin in Patients With Kidney Disease, With and Without Heart Failure	not in patients with HFpEF
5	Kusunose, K.	Effects of canagliflozin on NT-proBNP stratified by left ventricular diastolic function in patients with type 2 diabetes and chronic heart failure: a sub analysis of the CANDLE trial	not in patients with HFpEF
6	Tanaka, A.	Effect of canagliflozin on N-terminal pro-brain natriuretic peptide in patients with type 2 diabetes and chronic heart failure according to baseline use of glucose-lowering agents	not in patients with HFpEF
7	Verma, S.	Time to cardiovascular benefits of empagliflozin: a post hoc observation from the EMPA-REG OUTCOME trial	not in patients with HFpEF
8	Cahn, A.	Cardiovascular, Renal, and Metabolic Outcomes of Dapagliflozin Versus Placebo in a Primary Cardiovascular Prevention Cohort: Analyses From DECLARE-TIMI 58	not in patients with HFpEF
9	Ferreira, J.P.	Cardio/Kidney Composite End Points: A Post Hoc Analysis of the EMPA-REG OUTCOME Trial	not in patients with HFpEF
10	McMurray, J.	Effect of Dapagliflozin on Clinical Outcomes in Patients With Chronic Kidney Disease, With and Without Cardiovascular Disease	not in patients with HFpEF
11	Wheeler, D.C.	Effects of dapagliflozin on major adverse kidney and cardiovascular events in patients with diabetic and non-diabetic chronic kidney disease: a prespecified analysis from the DAPA-CKD trial	not in patients with HFpEF

12	Cahn, A.	Cardiorenal outcomes with dapagliflozin by baseline glucose-lowering agents: Post hoc analyses from DECLARE-TIMI 58	not in patients with HFpEF
13	Bohm, M.	Empagliflozin Improves Cardiovascular and Renal Outcomes in Heart Failure Irrespective of Systolic Blood Pressure	not in patients with HFpEF
14	Dewan, P.	Efficacy and safety of sodium-glucose co-transporter2inhibition according to left ventricular ejection fraction inDAPA-HF	not in patients with HFpEF
15	Fitchett, D.H.	Empagliflozin Reduces the Total Burden of Cardiovascular Events Including Recurrent Events in the EMPA-REG OUTCOME Trial	not in patients with HFpEF
16	Sindone, A.	Kidney function after initiation and discontinuation of empagliflozin in heart failure patients with and without type 2 diabetes: Insights from the emperial trials	not in patients with HFpEF
17	Docherty, K.F.	Effects of dapagliflozin in DAPA-HF according to background heart failure therapy	not in patients with HFpEF
18	Heerspink, H.J.L.	Dapagliflozin in patients with chronic kidney disease	not in patients with HFpEF
19	Wheeler, D.C.	Effects of dapagliflozin on kidney function, cardiovascular events, and all-cause mortality according to cause of kidney disease in the DAPA-CKD trial	not in patients with HFpEF
20	Nassif, M.E.	Main Results of The Empagliflozin Evaluation By Measuring Impact On Hemodynamics In Patients With Heart Failure Trial	not in patients with HFpEF
21	McMurray, J.J.V.	Dapagliflozin And Prevention of Adverse outcomes in Chronic Kidney Disease (DAPA-CKD)	not in patients with HFpEF
22	Li, J.	Effect of canagliflozin on total hospitalization for heart failure events in patients with type 2 diabetes and chronic kidney disease	not in patients with HFpEF

23	Arnott, C.	The effects of canagliflozin on heart failure and cardiovascular death by baseline participant characteristics: Analysis of the CREDENCE trial	not in patients with HFpEF
24	Inzucchi, S.E.	Consistent cardiovascular (CV) benefit of empagliflozin over the spectrum of CV risk factor control in EMPA-REG OUTCOME	not in patients with HFpEF
25	Mahaffey, K.W.	Canagliflozin and Cardiovascular and Renal Outcomes in Type 2 Diabetes Mellitus and Chronic Kidney Disease in Primary and Secondary Cardiovascular Prevention Groups: Results from the Randomized CREDENCE Trial	not in patients with HFpEF
26	McGuire, D.K.	Effects of empagliflozin on first and recurrent clinical events in patients with type 2 diabetes and atherosclerotic cardiovascular disease: a secondary analysis of the EMPA-REG OUTCOME trial	not in patients with HFpEF
27	Slomski, A.	Dapagliflozin Cut Risk of Worsening Heart Failure	not in patients with HFpEF
28	Jensen, J.	Metabolic effects of empagliflozin in heart failure: A randomized, double-blind, and placebo-controlled trial (Empire HF Metabolic)	not in patients with HFpEF
29	Sarraju, A.	Canagliflozin (CANA) reduces cardiovascular (CV) and renal events independent of baseline heart failure (HF): a CREDENCE secondary analysis	not in patients with HFpEF
30	Sarraju, A.	Effects of canagliflozin on cardiovascular, renal, and safety outcomes in participants with type 2 diabetes and chronic kidney disease according to history of heart failure: Results from the CREDENCE trial	not in patients with HFpEF
31	Magavern, E.	Effect of dapagliflozin on cardiovascular events in patients with type 2 diabetes	not in patients with HFpEF

32	Mcmurray. J.	The bexagliflozin efficacy and safety trial (BEST): a randomized, double-blind, placebo-controlled, phase IIII, clinical trial	not in patients with HFpEF
33	Januzzi, J.L.	Effects of Canagliflozin on Amino-Terminal Pro-B-Type Natriuretic Peptide: implications for Cardiovascular Risk Reduction	not in patients with HFpEF
34	Anker, S.D.	Baseline characteristics of patients with heart failure with preserved ejection fraction in the EMPEROR-Preserved trial	not in patients with HFpEF
35	Inzucchi, S.E.	Cardiovascular Benefit of Empagliflozin Across the Spectrum of Cardiovascular Risk Factor Control in the EMPA-REG OUTCOME Trial	not in patients with HFpEF
36	Wong, N.D.	Estimating the number of preventable cardiovascular disease events in the United States using the EMPA-REG OUTCOME trial results and National Health and Nutrition Examination Survey	not in patients with HFpEF
37	Bompoint, S.	Canagliflozin and renal outcomes in type 2 diabetes and nephropathy	not in patients with HFpEF
38	Sakai, T.	Sodium-glucose cotransporter 2 inhibitors reduce residual cardiovascular risk in heart failure with preserved ejection fraction	not in patients with HFpEF
39	McGuire, D.	Empagliflozin reduces the total burden of first and recurrent hospitalisations in patients with type 2 diabetes and established cardiovascular disease	not in patients with HFpEF
40	Inzucchi, S.E.	Consistent cardiovascular (CV) benefits from empagliflozin across the spectrum of CV risk factor control: Post hoc analysis from EMPA-REG OUTCOME	not in patients with HFpEF
41	Butler, J.	Empagliflozin Improves Kidney Outcomes in Patients With or Without Heart Failure	not in patients with HFpEF
42	Pellicori, P.	Early benefits of empagliflozin in patients with type 2 diabetes with heart failure are not offset by increased adverse events: results from the EMPA-REG OUTCOME trial	not in patients with HFpEF
43	Kosiborod, M.N.	Effect of Treatment on the Kansas City Cardiomyopathy Questionnaire (KCCQ) in the Dapagliflozin and	not in patients with HFpEF

		Prevention of Adverse-Outcomes in Heart Failure Trial (DAPA-HF)	
44	Scott, S.	Influence of Ejection Fraction on the Effect of Treatment in the Dapagliflozin and Prevention of Adverse-Outcomes in Heart Failure Trial (DAPA-HF)	not in patients with HFpEF
45	Figtree, G.A.	Effects of Canagliflozin on Heart Failure Outcomes Associated With Preserved and Reduced Ejection Fraction in Type 2 Diabetes Mellitus	not in patients with HFpEF
46	Wiviott, S.D.	The dapagliflozin effect on cardiovascular events (DECLARE)-TIMI 58 trial	not in patients with HFpEF
47	Rådholm, K.	Canagliflozin and Heart Failure in Type 2 Diabetes Mellitus: Results From the CANVAS Program	not in patients with HFpEF
48	Murakami, T.	Empagliflozin early reverses metabolic and cardiac disturbances in type-2 diabetics with chronic heart failure	not in patients with HFpEF
49	Inzucchi, S.E.	Consistent effect of empagliflozin on composite outcomes related to heart failure: results from EMPA-REG outcome	not in patients with HFpEF
50	Fitchett, D.	Heart failure outcomes with empagliflozin in patients with type 2 diabetes at high cardiovascular risk: results of the EMPA-REG OUTCOME® trial	not in patients with HFpEF
51	Inzucchi, S.E.	Empagliflozin and Cardiovascular Outcomes in Patients With Type 2 Diabetes Mellitus at High Cardiovascular Risk	not in patients with HFpEF
52	Zinman, B.	Empagliflozin, cardiovascular outcomes, and mortality in type 2 diabetes	not in patients with HFpEF
53	Soga, F.	Impact of Dapagliflozin on the Left Ventricular Diastolic Function in Diabetic Patients with Heart Failure Complicating Cardiovascular Risk Factors	not RCTs
54	Higashikawa, T.	Effects of Tofogliflozin on Cardiac Function in Elderly Patients With Diabetes Mellitus	not RCTs
55	Tanaka, H.	Positive effect of dapagliflozin on left ventricular longitudinal function for type 2 diabetic mellitus patients with chronic heart failure	not RCTs
56	Sakai, T.	Effect of sodium-glucose cotransporter 2 inhibitors on reducing residual cardiovascular risk and improving vascular endothelial function in patients with heart failure with preserved ejection fraction	not RCTs
57	Núñez, J.	Early effects of empagliflozin on exercise tolerance in patients with heart failure: A pilot study	not RCTs

58	Sakai, T.	Effect of sodium-glucose cotransporter 2 inhibitor on vascular endothelial function and diastolic function in patients with heart failure with preserved ejection fraction (hfpef)	not RCTs
59	Jose Morgado, J.	Sodium-glucose co-transporter-2 inhibitors as oral antidiabetic in patients with heart failure	not RCTs
60	Ou, Z.F.	Clinical efficacy of dapagliflozin in elderly female HF preserved ejection fraction patients with type 2 DM	not RCTs
61	Berg, D.	Mediation analysis for dapagliflozin and the reduction in hospitalization for heart failure in DECLARE-TIMI 58	abstracts of conference papers
62	Figtree, G.	Effects of canagliflozin on heart failure outcomes with and without preserved ejection fraction in type 2 diabetes: results from the CANVAS program	abstracts of conference papers
63	Soga, F.	Effect of dapagliflozin on left ventricular diastolic function of patients with type 2 diabetic mellitus with chronic heart failure	abstracts of conference papers
64	Sakai, T.	Right and left global longitudinal strain in heart failure with preserved ejection fraction predicts outcome for patients treated by sodium-glucose cotransporter 2 inhibitors	abstracts of conference papers
65	Sakai, T.	Effect of sodium-glucose cotransporter 2 inhibitor on diastolic function and global longitudinal strain (GLS) in patients with heart failure with preserved ejection fraction (HFpEF)	abstracts of conference papers
66	Branimir Kanazirev, B.	Reduction of NT-proBNP in patients with type 2 diabetes mellitus and heart failure with preserved ejection fraction in short-term treatment with empagliflozin on top of existing therapy	abstracts of conference papers
67	McGuire, D.K.	Effect of empagliflozin on cardiovascular events including recurrent events in the EMPA-REG OUTCOME trial	abstracts of conference papers
68	Sakai, T.	The evaluation of global longitudinal strain and the ratio of early mitral inflow velocity to global longitudinal strain rate in patients with heart failure with preserved ejection fraction treated by sodium-glucose cotransporter 2 inhibitors	abstracts of conference papers
69	Fitchett, D.	Effects of empagliflozin on cardiovascular mortality by prevalent or incident heart failure in the EMPA-REG OUTCOME trial	abstracts of conference papers
70	Nakashima, M.	Effects of luseogliflozin on estimated plasma volume in patients with heart failure with preserved ejection fraction	overlapped patient populations
71	Packer, M.	Effect of Empagliflozin on Worsening Heart Failure Events in Patients With Heart Failure and Preserved Ejection Fraction: EMPEROR-Preserved Trial	overlapped patient populations

72	Szarek, M.	Effect of Sotagliflozin on Total Hospitalizations in Patients With Type 2 Diabetes and Worsening Heart Failure: A Randomized Trial	overlapped patient populations
73	Cannon, C.P.	Cardiovascular outcomes with ertugliflozin in type 2 diabetes	overlapped patient populations
74	Savarese, G.	Empagliflozin in Heart Failure With Predicted Preserved Versus Reduced Ejection Fraction: Data From the EMPA-REG OUTCOME Trial	not in patients with HFpEF

Abbreviations: HFpEF , Heart failure with preserved ejection fraction; RCTs, randomized controlled trials; SGLT2, Sodium-Glucose cotransporter 2.

e Table 2. GRADE summary of evidence for effect of SGLT2 inhibitors on heart failure with preserved ejection fraction

Quality assessment							No. of patients		Effect	Quality	Importance
(outcomes) No of studies	Design	Risk of bias	Inconsistency	Indirectness	Imprecision	Other considerations	SGLT2 inhibitors	control	Absolute		
(Composite of first hospitalization for heart failure or cardiovascular death) 5	randomized trials	no serious risk of bias	no serious inconsistency	no serious indirectness	no serious imprecision	none	5046	4680	HR 0.78 (0.70 to 0.87)	⊕⊕⊕⊕ **high**	CRITICAL
(First hospitalization for heart failure) 3	randomized trials	no serious risk of bias	no serious inconsistency	no serious indirectness	no serious imprecision	none	4076	3727	HR 0.71 (0.62 to 0.83)	⊕⊕⊕⊕ **high**	CRITICAL
(Cardiovascular death) 3	randomized trials	no serious risk of bias	no serious inconsistency	no serious indirectness	no serious imprecision	none	4076	3727	HR 0.96 (0.82 to 1.13)	⊕⊕⊕⊕ **high**	CRITICAL
(Total hospitalizati	randomized trials	no serious risk of bias	no serious inconsistency	no serious indirectness	no serious imprecision	none	3516	3527	RR 0.75 (0.67 to 0.84)	⊕⊕⊕⊕ **high**	CRITICAL

on for heart failure) 4											
(All-cause mortality) 5	randomized trials	no serious risk of bias	no serious inconsistency	no serious indirectness	no serious imprecision	none	4316	3976	RR 0.99 (0.88 to 1.11)	⊕⊕⊕⊕ **high**	CRITICAL
(E/e’) 2	randomized trials	no serious risk of bias	no serious inconsistency	no serious indirectness	no serious imprecision	none	101	110	MD -1.22 higher (-2.29 to -0.15 higher)	⊕⊕⊕⊕ **high**	CRITICAL
(The change of NT-proBNP) 3	randomized trials	no serious risk of bias	no serious inconsistency	no serious indirectness	no serious imprecision	none	3110	3103	MD -26.60 higher (-61.2 to 7.99 higher)	⊕⊕⊕⊕ **high**	CRITICAL
(NT-proBNP at end of follow-up) 3	randomized trials	no serious risk of bias	no serious inconsistency	no serious indirectness	no serious imprecision	none	263	272	MD -8.51 higher (-33.19 to 16.16 higher)	⊕⊕⊕⊕ **high**	CRITICAL
(BNP) 2	randomized trials	no serious risk of bias	no serious inconsistency	no serious indirectness	no serious imprecision	none	204	202	MD -21.4 higher (-75.69 to 33.62 higher)	⊕⊕⊕⊕ **high**	CRITICAL
(6MWTD) 2	randomized trials	no serious risk of bias	no serious inconsistency	no serious indirectness	no serious imprecision	none	187	188	MD 14.99 higher (-4.60 to 34.60 higher)	⊕⊕⊕⊕ **high**	CRITICAL
(AEs) 7	randomized trials	no serious risk of bias	no serious inconsistency	no serious indirectness	no serious imprecision	none	3541	3541	RR 0.92 (0.88 to 0.97)	⊕⊕⊕⊕ **high**	CRITICAL

Abbreviations: AEs, adverse events; BNP, B-type natriuretic peptide; E/e’, the ratio of early mitral inflow velocity to mitral annular early diastolic velocity; GRADE, grading of recommendations assessment, development, and evaluation; HR, hazard ratios; MD, Mean Difference; NT-proBNP, N-terminal pro-B-type natriuretic peptide; RR, Risk Ratio; SGLT2, Sodium-Glucose cotransporter 2; 6MWTD, 6-minute walk test distance.

e Table 3. Effect of SGLT2 inhibitors compared with control on NT-proBNP, BNP and 6MWTD in patients with heart failure with preserved ejection fraction

	MD	95% CI		*P* value	I^2
		Lower Limit	Upper Limit		
The change of NT-proBNP	-26.60	-61.20	7.99	0.13	98%
NT-proBNP at end of follow-up	-8.51	-33.19	16.16	0.50	0%
BNP	-21.04	-75.69	33.62	0.45	72%
6MWTD	14.99	-4.60	34.60	0.13	87%

Abbreviations: BNP, B-type natriuretic peptide; CI, confidence intervals; MD, Mean Difference; NT-proBNP, N-terminal pro-B-type natriuretic peptide; SGLT2, Sodium-Glucose cotransporter 2; 6MWTD, 6-minute walk test distance.

e Figure 1. Sensitivity analysis of the composite endpoint in patients with HFpEF treated by SGLT2 inhibitors

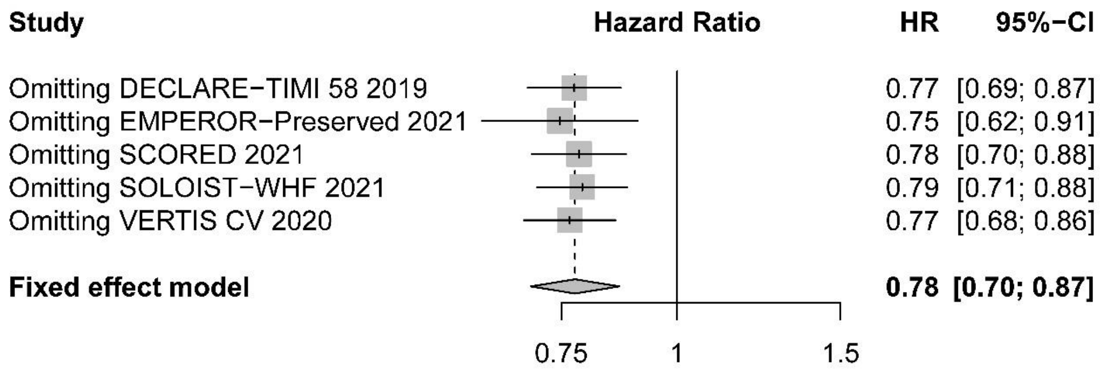

Supplement: Supplementary file 1 [file Data_Sheet_1.PDF]
